# The health of people classified as lesbian, gay and bisexual attending family practitioners in London: a controlled study

**DOI:** 10.1186/1471-2458-6-127

**Published:** 2006-05-08

**Authors:** Michael King, Irwin Nazareth

**Affiliations:** 1 Professor of Primary Care Psychiatry, Department of Mental Health Sciences, Royal Free and University College Medical School, University College London, Royal Free Campus, Rowland Hill Street, London NW3 2PF, UK; 2 Professor of Primary Care & Population Sciences & Director MRC General Practice Research Framework, Department of Primary Care and Population Sciences, Royal Free and University College Medical School, University College London (UCL), Rowland Hill Street, London NW3 2PF, UK

## Abstract

**Background:**

The morbidity of gay, lesbian or bisexual people attending family practice has not been previously assessed. We compared health measures of family practice attendees classified as lesbian, gay and bisexual.

**Methods:**

We conducted a cross-sectional, controlled study conducted in 13 London family practices and compared the responses of 26 lesbian and 85 bisexual classified women, with that of 934 heterosexual classified women and 38 gay and 23 bisexual classified men with that of 373 heterosexual classified men. Our outcomes of interest were: General health questionnaire; CAGE questionnaire; short form12; smoking status; sexual experiences during childhood; number of sexual partners and sexual function and satisfaction.

**Results:**

In comparison to people classified as heterosexuals: men classified as gay reported higher levels of psychological symptoms (OR 2.48, CI 1.05–5.90); women classified as bisexual were more likely to misuse alcohol (OR 2.73, 1.70–4.40); women classified as bisexual (OR 2.53, 1.60–4.00) and lesbian (OR 3.13, 1.41–6.97) and men classified as bisexual (OR 2.48, 1,04, 5.86) were more likely to be smokers and women classified as bisexual (OR 3.27, 1.97–5.43) and men classified as gay (OR 4.86, 2.28–10.34) were much more likely to report childhood sexual experiences in childhood. Psychological distress was associated with reporting sexual experiences in childhood in men classified as gay and bisexual and women classified as heterosexual. Men classified as bisexual (OR 5.00, 1.73–14.51) and women classified as bisexual (OR 2.88, 1.24- 6.56) were more likely than heterosexuals to report more than one sexual partner in the preceding four weeks. Lesbian, gay and bisexual classified people encountered no more sexual function problems than heterosexuals but men classified as bisexual (OR 2.74, 1.12–6.70) were more dissatisfied with their sex lives.

**Conclusion:**

Bisexual and lesbian classified people attending London general practices were more likely to be smokers and gay classified men were at increased risk of psychological distress in comparison to heterosexual classified people. Increased awareness of the sexuality of people seen in primary care can provide opportunities for health promotion.

## Background

Lesbian, gay and bisexual (LGB) people experience prejudice and discrimination [1] and may have higher rates of anxiety, depression, substance use disorders and suicidal behaviour than heterosexuals [2-5]. Furthermore, despite considerable data on the sexual behaviour of gay men, little is known about the prevalence of sexual dysfunction [6,7] and even less is known about sexual behaviour or dysfunction in lesbians [8] or bisexual people. Most research into the mental health of LGB people has been conducted in North America and many studies have included no comparison groups [7,9-11], recruited non-random samples [6,12] or applied unusual definitions of same sex attraction [4]. Few have distinguished gay and lesbian from bisexual people, classifying instead on any degree of same sex attraction [13], usually because of small samples. No previous study has assessed morbidity of gay, lesbian or bisexual people attending their family practitioners.

Although 5% of the British population is gay or lesbian [14] random sampling often achieves insufficient numbers [15] and/or such low percentages of LGB people that the research process becomes cumbersome and expensive [8]. We recruited people attending family practitioners in order to assess the importance of the sexual orientation of general practices attendees in London to their mental, physical and sexual function.

## Methods

### Participants and setting

Two London (the Camden and Islington and the Enfield and Haringey) local research ethical committees approved the study. We recruited people attending their primary care physicians. 95% of the people in the UK are registered at general practices that serve as the first point of contact with health services. We approached a group of research general practices in a defined area of north London. None of the practices approached served only a LGB population nor did they advertise or offer special health care to LGB people. We asked consecutive people aged 18 to 75 attending these general practices to participate in the study and in each practice recruited attendees over a 4–8 week period. Each person approached to participate in the study was given a detailed information sheet on the study procedures. Those consenting to take part were asked to complete a questionnaire in a private setting in the general practice as they waited to see the doctor [16].

## Materials

The questionnaire contained:

1. Standard demographic questions: on age, sex, ethnicity, civil status and current occupation.

2. A question on participants' sexual orientation using Kinsey ratings based on sexual experiences [17]. The respondent was asked to circle any number from 1 to 7 that corresponded to the statement that best described their sexual experiences. These were as follows: 1 entirely heterosexual; 2 largely heterosexual, but with some homosexual experience; 3 largely heterosexual, but considerable homosexual experience; 4 equally heterosexual and homosexual; 5 largely homosexual, but with considerable heterosexual experience; 6 largely homosexual, but with some heterosexual experience and 7 entirely homosexual.

3. Short Form 12 (SF-12): This is a well-validated quality of life questionnaire. We used the 12-item version of this questionnaire that produces separate physical and psychological well-being scores [18].

4. The General Health Questionnaire (GHQ): a measure of current mental health problems. Since its development, it has been extensively used in different settings and cultures [19-23]. The questionnaire was originally developed as a 60-item instrument but the GHQ-12, a shortened version of the questionnaire, has since been developed. The GHQ-12 is easy to complete and was designed to screen for psychological symptoms in the community. It is not a diagnostic instrument. There is evidence that the GHQ-12 is a consistent and reliable instrument when used in general population samples [24]. The GHQ asks whether the respondent has experienced a particular psychological symptom or behaviour recently. Each item is rated on a four-point scale (less than usual, no more than usual, rather more than usual, or much more than usual).

5. The four-item CAGE questionnaire [25], which is a known screening instrument for possible alcohol misuse. We also included one question on current consumption of cigarettes.

6. Questions about numbers of sexual partners in the preceding four weeks in order to assess risk of sexually transmitted infection.

7. Four screening questions about sexual experiences under the age of 16 years [26]. These concerned a) someone trying to or succeeding in having sexual intercourse with them b) touching, grabbing, kissing or rubbing up against them in public or private c) taking photographs of them naked or exhibiting parts of their body to them or performing a sex act in their presence and d) perpetrating oral sex or anal intercourse on them.

8. The brief sexual function questionnaire for men [27,28] and a modified version for women [29]. This questionnaire collected information on sexual activity (i.e. masturbation, oral sex and sexual intercourse) in the last month and also enabled us to make a sexual dysfunction diagnosis according to the 10^th ^Edition of the International Classification of Diseases [30] (lack/loss of sexual desire, sexual aversion, and dyspareunia in both sexes; arousal and orgasmic disorders and vaginismus in women and erectile and premature and retarded ejaculatory problems in men) and assessed their satisfaction with their sex life.

9. Total family practice consultations over the preceding two years were collected for those participants who allowed us access to their clinical records.

### Definition of sexual orientation and physical, psychological and sexual problems

a) *Sexual Orientation: *There is still no universal agreement on how to define sexual orientation [31]. Thus we approached our analysis on the assumption that society is more accepting of people who report largely heterosexual, rather than largely homosexual, experiences. Hence we classified people responding to rating 1 on our sexual experiences scale as unequivocally heterosexual. Similarly, we assumed that participants who indicated their experiences had been largely heterosexual or equally heterosexual and homosexual (ratings 2 to 4) were *primarily *identifying with heterosexuality (despite sexual experiences with both sexes) and classified them as bisexual. Finally we assumed that those who indicated that homosexual experiences were a large or entire part of their sexual lives (ratings 5 to 7) were likely to be primarily gay or lesbian and were classified accordingly.

b) *Poor physical function (quality of life) *was defined as those scoring below the 25^th ^centile of the physical function subscale score of the short form12 questionnaire.

c) *Psychological distress *The GHQ-12 provides a total score of 36, based on the Likert scoring of 0-1-2-3 or 12 based on a bi-modal (0-0-1-1). We used the latter as it is a more common scoring for psychological symptoms and defined those scoring three or more as likely to have significant psychological distress [20].

d) *Problems with alcohol use *were defined as those scoring two or more on the CAGE questionnaire.

e) *Current smokers *were defined as those that admitted to being smokers at the time of interview.

f) *Sexual experiences in childhood *were defined as unequivocal in participants who gave affirmative answers to at least three of the four screening questions.

g) *Sexual function problems*: were defined using responses from the brief sexual function questionnaires. In order to meet conservative criteria for a clinical ICD-10 diagnosis (F52.0 to F52.6) the problem needed to be present all or almost all of the time for each diagnosis [16]. However, to account for variation in sexual practices in same sex relationships, we widened the sexual situations in which arousal and orgasmic disorders might occur to include masturbation and oral sex. As previously described [16], we examined any sexual problem reported without requiring that the participant have a sexual partner or report sexual intercourse in the preceding four weeks.

h) *Consultations: *people were classed as high consulters if their total consultation rate over the previous two years exceeded the 75^th ^centile for the study population.

### Analysis

We examined the data using descriptive statistics. Differences in the demographic, sexual activity, and health outcome data between gay/lesbian, bisexual and heterosexual classified people were examined using the Chi squared statistic for dichotomous and analysis of variance for continuous data. We then explored the influence of age, civil status, ethnicity and current employment on sexual orientation and the primary outcomes of interest. These were physical quality of life (as measured on SF-12) and psychological distress (on the GHQ-12), sexual function, number of sexual partners in the previous month, childhood sexual experiences, CAGE scores, smoking status and consultation rates. Only civil status (married or living with a partner versus the remainder) and ethnicity (white versus non white) were found to be associated both with sexuality and the health outcomes at a significance level of 10% or less. Thus, we adjusted the analysis of each health factor against our classification of sexual orientation for each of these confounders by fitting logistic models and comparing their fit using the likelihood ratio. We report the p values to indicate whether the adjustments made a significant difference to the model. People in the heterosexual group were used as the index population on the grounds of their overwhelming majority. Each multivariate analysis was conducted separately for men and women. We analysed the data using SPSS version 10 and Stata version 7.

## Results

### Response rates and sexuality

We approached 37 North London general practices situated in areas of high, medium and low socio-economic deprivation. Thirteen practices (35%) with 55 doctors took part. We found no significant differences in Jarman's underprivileged area scores (that indicate the extent of socio-economic deprivation) between participating and non-participating practices. 1512 (71.6%) people (1065 women and 447 men) of the 2121 eligible general practice attendees participated but only 771 women and 307 men consented to access to their clinical records [16]. Twenty women (2.6%) and 13 (4.2%) men did not answer the question on sexual orientation while 85 (8%) women and 23 (5%) men were classified as bisexual and 38 (9%) men and 26 (3%) women as gay or lesbian (tables 1, 2). White male participants were more likely to be classified as gay and white female participants were more likely to be classified as bisexual while those classified as lesbian and gay were most likely to be cohabiting. Men and women classified as bisexual were more sexually active than those classified as gay or heterosexual.

### Associations between health, sex and sexual orientation

There were significant differences between the men classified as gay, bisexual and heterosexual on: mental health problems based on the GHQ-12; having more than one sexual partner in the previous month; reporting sexual experiences in childhood and satisfaction with their sex lives (table 3). Significant differences were observed between women classified as lesbian, bisexual and heterosexual on: current smoking; alcohol misuse based on CAGE scores; having more than one sexual partner in the previous month and having had sexual experiences in childhood (table 4). There were no differences in consultation rates between the sexual orientation groups for the 71% of people who allowed us access to their records.

### Independent factors associated with health and well-being

After adjustment for civil and ethnic status, men classified as bisexual were more likely than those classified as heterosexual to be current smokers (OR 2.48), to report having had more than one sexual partner in the last four weeks (OR 5.0) and to be dissatisfied with their sex lives (OR 2.74) (table 3). Men classified as gay were more likely than those classified as heterosexual (OR 2.52) to score above the threshold of the general health questionnaire indicating current psychological distress and to report sexual experiences in childhood (OR 4.86) (table 3). Women classified as bisexual (OR 2.53) and lesbian (OR 3.13) were more likely to be current smokers than those classified as heterosexual (table 4). Women classified as bisexual were significantly more likely than those classified as heterosexual (OR 2.73) to record a positive CAGE score indicating possible alcohol misuse, to have had more than one sexual partner in the last four weeks (OR 2.85) and to report sexual experiences in childhood (OR 3.27) (table 4).

### Sexual experiences in childhood and current pyshcolgical distress

Sexual experiences in childhood and high GHQ-12 scores appeared to occur together in gay and bisexual classified men (table 3) and so we explored their association further. To do this we compared heterosexual classified participants with gay and bisexual classified participants combined. This revealed associations only for gay and bisexual men and heterosexual women. Thirty-six per cent (18/50) of heterosexual classified men who reported childhood sexual experiences had GHQ-12 scores over 2 compared to 34% (107/315) of such men who did not report childhood experiences. Similarly, 48% (15/31) of lesbian and bisexual classified women who reported childhood experiences had GHQ-12 scores over 2 compared to 44% (35/79) of such women who did not report childhood experiences. In contrast, 65% (15/23) of gay and bisexual classified men who reported childhood sexual experiences had high GHQ-12 scores compared to 45% (n = 9/20) who did not report childhood experiences (Chi2 = 6.16, P = 0.013). And similarly, 51% (57/112) of heterosexual classified women who reported childhood sexual experiences had high GHQ-12 scores compared to 38% (306/808) who did not report such experiences (Chi2 = 6.98, P = 0.008).

## Discussion

### Main findings

In comparison to their heterosexuals counterparts: 1) women classified as lesbian were more likely to be smokers; 2) men classified as gay had higher levels of psychological symptoms and were more likely to report childhood sexual experiences; 3) women classified as bisexual were more likely to misuse alcohol, to be smokers, to report more than one sexual partner in the preceding four weeks and to report childhood sexual experiences; 4) men classified as bisexual were more likely to report more than one sexual partner in the preceding four weeks and to be dissatisfied with their sex lives. Reporting childhood sexual experiences was associated with adult psychological distress in gay and bisexual classified men and heterosexual classified women.

### Strengths and limitations of the study

To our knowledge this is the first European study in which the mental and physical health of people of a range of sexual orientation, attending family practitioners has been compared. Consecutive recruitment meant that our samples of gay and bisexual people were comparatively small and led to relative uncertainty in our estimates of odds ratios. In keeping with the higher rate of attendance of women compared with men in UK general practice [32], in this study we recruited just over twice the numbers of women rather than men. We did not find any differences in the consultation rates of attendees classified as gay, lesbian, bisexual or heterosexual. Nevertheless, it is still possible that differences in help seeking behaviour between men and women and between people classified as gay, lesbian and bisexual would make it difficult to generalise our finding to either sex or people classified as gay, lesbian or bisexual as a whole. Recruiting general practice attendees also means that the prevalence of physical or psychological difficulties may have been higher than in the general population, given that participants would often have been seeking help for medical or social problems. Our results are hence limited to the people recruited to our study and may not represent that of the UK or Europe as a whole.

We used a definition of sexuality based on criteria developed for this study. There is little consensus on how to measure sexual orientation. Several issues should be considered. Firstly, sexual responsiveness to others of the same sex, like most human traits is believed to be continuously distributed in the population [17,33]. Secondly, it may be incorrect to presume that such traits are stable within each person over time [34]. Thirdly, conflating any same-sex experiences with a categorization of the person as homosexual may present limitations when defining sexuality. Lastly, defining sexuality solely on the basis of sexual experience [17] may exclude people who fantasize about sex with others of the same sex but never have sexual contact [35]. Modern concepts of sexual orientation consider personal identification, sexual behaviour and sexual fantasy [36]. Few studies, however, utilise all these three definitions in arriving at a composite categorization of sexuality. One widely established definition is a person "with an orientation towards people of the same gender in sexual behaviour, affection, or attraction, and/or self-identity as gay/lesbian or bisexual" [37]. Using these types of definition, there is evidence that at least five per cent of people in western countries are gay or bisexual [14,34-38]. In our study classification of sexuality was limited by the Kinsey classification for which no time frame was applied. Moreover the classification of gay, bisexual and heterosexual as applied to people recruited in our study has not been previously used in similar population and it is possible that a different classification using the Kinsey scale might have yielded different results. Nevertheless, based on the categorization of sexual orientation used in this study, prevalence estimates of gay, lesbian and bisexual people in our sample closely matched that reported from London in a larger UK wide population survey [14].

There were also limitations in the way some data were collected. We assessed sexual behaviour using standardised questionnaires that were not explicit in their definition of sexual intercourse [27-29]. Many people (gay or straight) regard sexual intercourse differently and do not always realise that it means penetrative (vaginal or anal) sex. Furthermore, for gay men receptive anal intercourse can be regarded as "sexual intercourse" more often than insertive anal intercourse. At first sight our figures for sexual intercourse in gay classified men may seem low and those for lesbian classified woman high. However, most of our evidence on the prevalence of anal intercourse in gay men arises from particular populations, such as younger age groups, men attending clubs and bars or those living in gay neighbourhoods. Studies using diary records show that reported rates of intercourse depend on whether not the man has a regular same-sex partner and if so whether the relationship is open or closed. Those in closed relationships and those without regular partners may have relatively low rates of penile-anal intercourse [39]. Given the wide age range in our sample and the fact that these men were not selected on any sexual risk grounds, it is not surprising that only 35% of gay men reported sexual intercourse in the preceding 4 weeks. Moreover, vaginal penetrative practices using sex toys and fingers or hands are commonly reported by lesbian and bisexual women [40,41].

### Mental health of gay men and lesbians

Our finding of higher rates of common psychological symptoms of depression and anxiety, in gay classified men accords with those of a recent study of the mental health of gay men and lesbians in England and Wales in which a large sample was recruited by snowball sampling [12]. There are a number of reasons why gay people may be more likely to report psychological difficulties, which include difficulties growing up in a world orientated to heterosexual norms and values and the negative influence of social stigma against homosexuality. In addition, the gay commercial world in which some men and women may participate to find partners and friends may make misuse of alcohol and cigarettes more likely [3,12]. The former in particular can have adverse effects on mental well-being. Finally, our results add to evidence that sexual experiences in childhood in men classified as gay or bisexual may play a role in adult psychological adjustment [42,43]. Why this is not the case in lesbian and bisexual classified women requires further exploration.

### Mental health of bisexual people

Much less is known about how bisexual, as opposed to gay people, accept their sexual orientation or whether the possibility of having partners of either sex introduces difficulties. Although there is anecdotal evidence that bisexuality is regarded negatively by gay and lesbian political and social groups, it may present to people living in a predominantly heterosexual world as being of a more acceptable social status than that of a gay or lesbian identity. We found, however, little difference in psychological distress between people classified as bisexual and heterosexual, despite the finding that women classified as bisexual reported more sexual experiences in childhood than heterosexual classified women. This finding has also been reported in a population-based study in North American, where bisexual (and lesbian) women had higher lifetime rates of substance misuse and coerced sex than heterosexual women [44].

### Physical and sexual health

There were no differences between people classified as gay and heterosexual in terms of physical functioning based on a quality of life measure. People classified as bisexual on the other hand were more likely to be smokers raising concern about smoking related diseases such as cancers, cardiovascular diseases and chronic obstructive airway diseases. There is now good data on smoking among gay, lesbian and bisexual people. Data from population based health surveys and random student population samples in North America have suggested high rates of smoking in LGB people [38]. Men and women classified as bisexual were also more likely than heterosexuals to report contact with two or more sexual partners in the preceding month, potentially increasing their likelihood of acquiring sexually transmitted infections. For women classified as bisexual this combination with alcohol misuse could also place them at a greater risk of sexually transmitted infections. There is good evidence that risky sexual behaviour is increasing among men and women but comparative data on sexual behaviour of different sexual orientations has to our knowledge not been previously reported [14].

### Family practice

There is varying evidence on the use of family practice by lesbian, gay and bisexual people. We have previously reported that LGB people consult general practitioners for emotional reasons more often than heterosexuals [12]. However, other research in the UK suggests that LGB people may avoid primary medical care [49] and those that do attend may not reveal their sexual orientation [50]. Awareness of a patient's sexual orientation may alert the family practitioner to potential difficulties, particularly with regard to alcohol misuse and smoking and to some extent sexual behaviour. There is a clear need for awareness on the part of family practitioners and openness on the part of patients about sexual orientation. This would allow practice staff adequate opportunities to monitor the smoking status, alcohol use, mental health, current numbers of sexual contacts and childhood sexual experiences in people of range of sexual orientation attending family practice. People at risk could then be offered early interventions to minimise their chances of developing more advanced illnesses.

## Conclusion

Bisexual and lesbian classified people attending London general practices were more likely to be smokers and gay classified men were at increased risk of psychological distress when compared to people classified as heterosexual. Sexual experiences in childhood were associated with current psychological distress in heterosexual classified women and in gay and bisexual classified men but this was not the case for non-heterosexual women or heterosexual classified men. Increased awareness of the sexuality of people seen in primary care can provide opportunities for health promotion.

## Competing interests

The author(s) declare that they have no competing interests.

## Authors' contributions

MK and IN conceived the idea, obtained funding for the study, analysed the data and wrote the paper. MK and IN are guarantors for the study.

## Pre-publication history

The pre-publication history for this paper can be accessed here:



## Figures and Tables

**Table 1:** Demographic details and sexual activity of men

	Heterosexual classified N = 373	Bisexual classified N = 23	Gay classified N = 38
Age – Mean(SD)^M = 6^	35.9 (12.3)	37.1 (12.0)	35.9 (12.3)
			

Ethnic status^M = 6^			
White#	268 (72.6)	19 (82.6)	34 (94.4)
Black	77 (20.9)	1 (4.4)	2 (5.6)
South Asian	8 (2.2)	2 (8.7)	0
Others	16 (4.4)	1 (4.4)	0

Occupation^M = 6^			
Full/part time work	252 (68.3)	14 (60.9)	28 (77.8)

Civil Status^M = 6^			
Married	107 (28.7)	7 (30.4)	1 (2.6)
Co-habiting	85 (22.8)	6 (26.1)	12 (31.6)

Sexual activity (at least once/last month)			
Masturbation* ^M = 6^	225 (61.1)	19 (82.6)	29 (78.4)
Oral Sex^M = 3^	193 (52.0)	13 (56.5)	21 (56.8)
Sex Intercourse** ^M = 5^	263 (71.3)	18 (78.3)	13 (35.1)

No. of partners^M = 1^			
None	102 (27.4)	5 (21.7)	12 (31.6)
One	243 (65.3)	12 (52.2)	18 (47.4)
2–10***	27 (7.3)	6 (26.1)	8 (21.1)

M = Number of non responders

N = column percent,

*p = 0.018,**p = 0.0005, ***p = 0.002

# white versus non-white p = 0.011

**Table 2:** Demographic details and sexual activity of women

	Heterosexual classified N = 934	Bisexual classified N = 85	Lesbian classified N = 26
Age –Mean(SD)^M = 6^	33.1(12.1)	31.4 (11.7)	32.7(11.7)

Ethnic status^M = 6^			
White#	684 (73.6)	72 (85.7)	19 (73.6)
Black	178 (19.1)	5 (6.0)	6 (23.1)
South Asian	32 (3.4)	2 (2.4)	0
Others	36 (3.9)	5 (6.0)	1 (3.9)

Occupation^M = 6^			
Full/part time work	523 (56.2)	45 (54.2)	12 (46.2)

Civil Status^M = 6^			
Married	243 (26.0)	6 (7.1)	1 (3.8)
Co-habiting*	228 (24.4)	22 (25.9)	15 (57.7)

Sexual activity (at least once/last month)			
Masturbation** ^M = 6^	293 (31.4)	58 (69.1)	9 (34.6)
Oral Sex^M = 3^	359 (38.6)	39 (46.4)	12 (46.2)
Sex Intercourse^M = 5^	626 (67.1)	50 (59.5)	13 (50.0)

No. of partners^M = 1^			
None	276 (29.7)	27 (32.8)	10 (38.5)
One	243 (65.3)	50 (58.8)	15 (57.7)
2–10 ***	28 (3.0)	8 (9.4)	1 (3.9)

M = Number of non responders

N = column percent,

*p = 0.004, **p = 0.0005,

*** 2–10 partners vs. the rest p = 0.014

# white versus non-white p = 0.04

**Table 3:** Distribution of health and health behaviours for men

Variable	Heterosexual classified N = 373	Bisexual classified N = 23	Gay classified N = 38	Chi2, p value
Smoking^M = 4^	144 (39)	14 (61)	19 (50)	5.61 P = 0.06
SF12 physical scale score less than 25^th ^centile^M = 23^	97 (27)	3 (16)	13 (35)	2.39 P = 0.30
GHQ high scorer	129 (35)	6 (26)	22 (58)	9.19 P = 0.01
CAGE high scorer	119 (32)	9 (39)	13 (34)	0.57 P = 0.75
More than 1 sexual partner in previous month^M = 1^	27 (7)	6 (26)	8 (21)	15.48 P = 0.00
Any sexual function problem^M = 8^	167 (46)	14 (61)	21 (55)	3.01 P = 0.22
Childhood sexual experiences score 3 or more^M = 9^	50 (14)	6 (26)	17 (46)	25.91 P = 0.00
Dissatisfied with sex life^M = 2^	218 (59)	8 (35)	16 (42)	8.33 P = 0.02
Consultations over 75^th ^centile^M = 133^	54 (21)	3 (20)	8 (29)	0.89 P = 0.64

M = Number of non responders

N = column per cent

**Table 4:** Distribution of health and health behaviours for women

Variable	Heterosexual classified N = 934	Bisexual classified N = 85	Lesbian classified N = 26	Chi2, p value
Smoking^M = 10^	294 (32)	48 (57)	15 (58)	28.27 P = 0.00
SF12 physical scale score less than 25^th ^centile^M = 20^	233 (25)	23 (28)	6 (25)	0.29 P = 0.86
GHQ high scorer	371 (40)	40 (47)	11 (42)	1.78 P = 0.41
CAGE high scorer	164 (18)	35 (41)	8 (31)	29.37 P = 0.00
More than 1 sexual partner in previous month^M = 4^	28 (3)	8 (9)	1 (4)	9.32 P = 0.01
Any sexual function problem^M = 2^	567 (61)	51 (60)	14 (54)	0.53 P = 0.77
Childhood sexual experiences score 3 or more^M = 15^	112 (12)	27 (32)	4 (15)	25.72 P = 0.00
Dissatisfied with sex life^M = 10^	462 (50)	46 (55)	16 (62)	1.98 P = 0.37
Consultations over 75^th ^centile^M = 287^	186 (27)	13 (22)	5 (25)	0.83 P = 0.66

M = Number of non responders

N = column per cent

**Table 5:** Health comparisons in heterosexual, bisexual and gay classified men

Variable	Predictor	number	Odds ratio for increased risk	LR chi2	p-value
Smoking	Bisexual	424	**2.47 (1.04, 5.86)**		
	Gay		1.42 (0.71, 2.83)		
	Bisexual		**2.48 (1.05, 5.90)**		
	Gay		1.36 (0.67, 2.73)		
	White		1.06 (0.67, 1.67)		
	Civil status#		0.81 (0.55, 1.20)	1.15	0.56

SF12 physical scale score less than 25^th ^centile	Bisexual*	405	0.49 (0.14, 1.75)		
	Gay		1.57 (0.76, 3.24)		
	Bisexual		0.49 (0.14, 1.72)		
	Gay		1.49 (0.71, 3.11)		
	White		1.21 (0.72, 2.04)		
	Civil status		0.93 (0.60, 1.44)	0.63	0.730

GHQ high scorer	Bisexual	405	0.66 (0.25, 1.71)		
	Gay		**2.60 (1.30, 5.23)**		
	Bisexual		0.66 (0.26, 1.73)		
	Gay		**2.52 (1.25, 5.11)**		
	White		0.97 (0.61, 1.55)		
	Civil status		0.78 (0.52, 1.15)	1.56	0.46

CAGE high scorer	Bisexual	428	1.38 (0.58, 3.30)		
	Gay		0.95 (0.45, 1.99)		
	Bisexual		1.35 (0.57, 3.23)		
	Gay		0.85 (0.40, 1.80)		
	White		1.46 (0.89, 2.40)		
	Civil status		0.80 (0.53, 1.21)	3.39	0.18

More than 1 sexual partner in previous month	Bisexual	427	**4.45 (1.62, 12.24)**		
	Gay		**3.05 (1.22, 7.60)**		
	Bisexual		**5.00 (1.73, 14.51)**		
	Gay		2.35 (0.91, 6.06)		
	White		1.74 (0.69, 4.41)		
	Civil status		0.24 (0.11, 0.53)	16.22	0.0003

Any sexual function problem	Bisexual	421	1.82 (0.77, 4.30)		
	Gay		1.46 (0.73, 2.91)		
	Bisexual		1.86 (0.78, 4.41)		
	Gay		1.47 (0.73, 2.97)		
	White		0.86 (0.55, 1.35)		
	Civil status		0.86 (0.59, 1.27)	0.97	0.62

Childhood sexual experiences score of 3 or more	Bisexual	419	2.25 (0.84, 5.97)		
	Gay		**4.77 (2.29, 9.95)**		
	Bisexual		2.17 (0.81, 5.81)		
	Gay		**4.86 (2.28,10.34)**		
	White		1.31 (0.67, 2.54)		
	Civil status		1.46 (0.85, 2.50)	2.57	0.28

Dissatisfied with sex life	Bisexual	426	**2.62 (1.08, 6.34)**		
	Gay		1.75 (0.88, 3.48)		
	Bisexual		**2.74 (1.12, 6.70)**		
	Gay		1.56 (0.77, 3.15)		
	White		1.11 (0.70, 1.76)		
	Civil status		0.52 (0.35, 0.77)	11.2	0.004

Consultations over 75^th ^centile	Bisexual	300	0.94 (0.26, 3.44)		
	Gay		1.50 (0.63, 3.60)		
	Bisexual		1.00 (0.27, 3.71)		
	Gay		1.53 (0.63, 3.73)		
	White		0.75 (0.39, 1.45)		
	Civil status		0.83 (0.48, 1.45)	1.09	0.58

Odd Ratio below the line in each row are adjusted for civil and ethnic status

LR = Likelihood ratio * Heterosexual used as reference category OR 1.0 # married/cohabiting

**Table 6:** Health comparisons in heterosexual, bisexual and lesbian classified women

Variable	Variable	number	Odds ratio for increased risk	LR chi2	p-value
Smoking	Bisexual	1030	**2.85 (1.81, 4.49)**		
	Lesbian		**2.92 (1.32, 6.43)**		
	Bisexual		**2.53 (1.60, 4.00)**		
	Lesbian		**3.13 (1.41, 6.97)**		
	White		1.63 (1.19, 2.24)		
	Civil status		0.64 (0.49, 0.83)	19.02	0.0001

SF12 physical scale score less than 25^th ^centile	Bisexual	1022	1.14 (0.69, 1.89)		
	Lesbian		0.98 (0.38, 2.45)		
	Bisexual		1.07 (0.64, 1.78)		
	Lesbian		0.98 (0.38, 2.50)		
	White		1.53 (1.09, 2.16)		
	Civil status		0.91 (0.68, 1.21)	6.47	0.039

GHQ high scorer	Bisexual	1039	1.33 (0.85, 2.08)		
	Lesbian		1.12 (0.51, 2.47)		
	Bisexual		1.24 (0.79, 1.95)		
	Lesbian		1.15(0.52, 2.53)		
	White		1.26 (0.95, 1.69)		
	Civil status		0.81 (0.63, 1.04)	4.95	0.08

CAGE high scorer	Bisexual	1039	**3.17 (1.99, 5.06)**		
	Lesbian		2.07 (0.89, 4.85)		
	Bisexual		**2.73 (1.70, 4.40)**		
	Lesbian		2.23 (0.94, 5.30)		
	White		2.35 (1.54, 3.59)		
	Civil status		0.61 (0.45, 0.85)	24.95	0.0000

More than 1 sexual partner in previous month	Bisexual	1035	**3.37 (1.48, 7.65)**		
	Lesbian		1.28 (0.17, 9.79)		
	Bisexual		**2.85 (1.24, 6.56)**		
	Lesbian		1.40 (0.18, 10.80)		
	White		1.48 (0.64, 3.45)		
	Civil status		0.44 (0.21, 0.90)	5.96	0.05

Any sexual function problem	Bisexual	1037	0.95 (0.60, 1.49)		
	Lesbian		0.75 (0.34, 1.64)		
	Bisexual		0.98 (0.62 1.55)		
	Lesbian		0.72 (0.33, 1.59)		
	White		1.16 (0.87, 1.54)		
	Civil status		1.36 (1.06, 1.75)	7.26	0.03

Childhood sexual experiences score of 3 or more	Bisexual	1025	**3.43 (2.08, 5.56)**		
	Lesbian		1.32 (0.45, 3.89)		
	Bisexual		**3.27 (1.97, 5.43)**		
	Lesbian		1.32 (0.44, 3.92)		
	White		1.46 (0.93, 2.30)		
	Civil status		0.97 (0.67, 1.39)	2.90	0.23

Dissatisfied with sex life	Bisexual	1030	1.22 (0.78, 1.90)		
	Lesbian		1.61 (0.72, 3.58)		
	Bisexual		1.08 (0.68, 1.71)		
	Lesbian		1.76 (0.78, 3.98)		
	White		0.97 (0.73, 1.29)		
	Civil status		0.48 (0.38, 0.62)	33.04	0.0000

Consultations over 75^th ^centile	Bisexual	755	0.75 (0.40, 1.42)		
	Lesbian		0.88 (0.32, 2.47)		
	Bisexual		0.77 (0.40, 1.47)		
	Lesbian		0.87 (0.31, 2.47)		
	White		0.98 (0.66, 1.45)		
	Civil status		1.16 (0.84, 1.61)	0.81	0.67

Odd ratios below the line in each row are adjusted for civil and ethnic status

LR = likelihood ratio Heterosexual used as reference category OR 1.0 # married/cohabiting
